# Brow and Eyelid Rejuvenation: Trends from the 100 Most Cited Articles over 30 Years

**DOI:** 10.3390/medicina59020230

**Published:** 2023-01-26

**Authors:** Doga Kuruoglu, Cristina A. Salinas, Daniel S. Kirk, Chin-Ho Wong, Basel A. Sharaf

**Affiliations:** 1 Division of Plastic Surgery, Department of Surgery, Mayo Clinic, Rochester, MN 55905, USA; 2 Division of Oral and Maxillofacial Surgery, Department of Surgery, Mayo Clinic, Rochester, MN 55905, USA; 3 W Aesthetic Plastic Surgery, Department of Plastic Reconstructive and Aesthetic Surgery, Singapore General Hospital, The Centre for Facial Plastic Surgery, Singapore 329563, Singapore

**Keywords:** blepharoplasty, browlift, eyelid anatomy, facelift, facial aging, fat compartment, fat graft, periorbital rejuvenation

## Abstract

*Background and Objective*: Various periorbital rejuvenation techniques have been introduced over the last 3 decades. This study highlights important milestones in the evolution of periorbital rejuvenation surgery by identifying the 100 most-cited articles in this field. *Material and Methods:* The Web of Science citation index was used to identify the 100 most-cited articles concerning periorbital rejuvenation. Articles published in English from January 1989–April 2020 describing periorbital rejuvenation-related surgical techniques, facial aging, and anatomy were included. The terms “lower blepharoplasty”, “upper blepharoplasty”, “browlift”, “browplasty”, “endobrow lift”, “endoscopic brow”, “Foreheadplasty”, “lower eyelid anatomy”, “upper eyelid anatomy”, “forehead lift”, “eyelid rejuvenation”, “canthopexy”, “canthoplasty”, “eyelid fat pad”, “orbital fat pad”, “tear trough”, and “eyelid bags” were entered into the citation search. Web of Science Core Collection was the database used for the search. A manual review of the initial 159 studies was performed. Articles describing reconstructive or non-invasive techniques, injectable fillers, lasers, and neurotoxins were excluded. Of the 100 most-cited articles, the publication year, specialty journal, the corresponding author’s primary specialty, the focus of the article, the corresponding author’s country of residence, the type of study, and the level of evidence were analyzed. *Results*: The mean number of citations per article was 75 ± 42. There were more articles published from 1989–1999 (*n* = 53) than later decades. Most articles originated from the USA (*n* = 82) and were published in plastic surgery journals (*n* = 81). Plastic surgery was the primary specialty of the corresponding authors (*n* = 71), followed by oculoplastic surgery (*n* = 22). Most articles (*n* = 69) reported on surgical techniques. Of the clinical studies (*n* = 69), 45 (79%) provided level IV evidence. *Conclusions:* Of the 100 most-cited studies on periorbital rejuvenation, studies focusing on periorbital anatomy, aging, and surgical techniques comprised the most-cited publications. An anatomically based approach accounting for age-related changes in the periorbital structures is paramount in the field of contemporary periorbital rejuvenation.

## 1. Introduction

The human face is composed of layers that play a role in facial appearance and aging. These layers are arranged in five lamellar components that are clearly defined in the scalp and range from superficial to deep, as follows: skin, subcutaneous tissue, the musculo-aponeurotic layer (superficial musculoaponeurotic system—SMAS), loose areolar tissue, and the deep fascia or periosteum. Facial rejuvenation is based on the manipulation and re-draping of these tissue layers as well as specific anatomic attachments including ligaments, adhesions, and septa. 

Rejuvenation, a word originating from the Latin words “*re*” and “*juvenis*”, meaning “*young again*”, has always been a historic interest in various cultures and civilizations. The first historically reported procedures for periorbital rejuvenation date back to the first century and stem from Aulus Cornelius Celsus’s seventh book of his encyclopedia *De Medicina Ovto Libri* [1]. Later accounts of the cauterization of excess eyelid skin by Arabian surgeons were reported in the 10th century [2]. In the 17th century, Ambroise Pare [3] performed corrections of excess eyelid skin. In 1896, Fuchs [4] introduced the word “blepharochalasis” to describe the thin, wrinkled eyelids of a teenager. In 1924, Bourguet [5] described transconjunctival lower eyelid blepharoplasty. In 1931, Claoué [6] and Passot [7] described the removal of the bulging fat pad in the lower eyelid, which was previously described by Bourguet through a transconjunctival approach. In 1947, Pierce et al. [8] described a technique of excising the corrugator supercilii muscle to treat frowning in a teacher. This was followed by Bames [9] in 1957, who described a technique for skin ellipse excision directly above the brow and the complete resection of the corrugator supercilii muscle to correct brow ptosis and frowning, respectively. In the same decade, Castañares [10] published a landmark article in which he described the orbital fat pad compartments in detail. He based his new blepharoplasty technique on this anatomical description, which became the basis for modern blepharoplasty. Subsequent reports in the late 1960s and 1970s by Rhees [11], Loeb [12], and Furnas [13] described the excision of redundant orbicularis oculi muscle tissue and bulging fat pads, emphasizing the importance of conservative resection.

An in-depth understanding of the periorbital anatomy and corresponding sub-units is paramount when planning rejuvenation in this area [14,15,16]. As various rejuvenation techniques have been introduced over the last 3 decades and our understanding of periorbital anatomy and aging has evolved, we aim to highlight the important milestones in our understanding of periorbital aging and the anatomy and evolution of periorbital rejuvenation surgical techniques. The 100 most-cited articles on aesthetic periorbital surgery were identified and presented in chronological order to serve as a resource for surgeons and trainees. Current approaches to eyelid and eyebrow rejuvenation are also presented.

## 2. Methods

**Figure 1:**
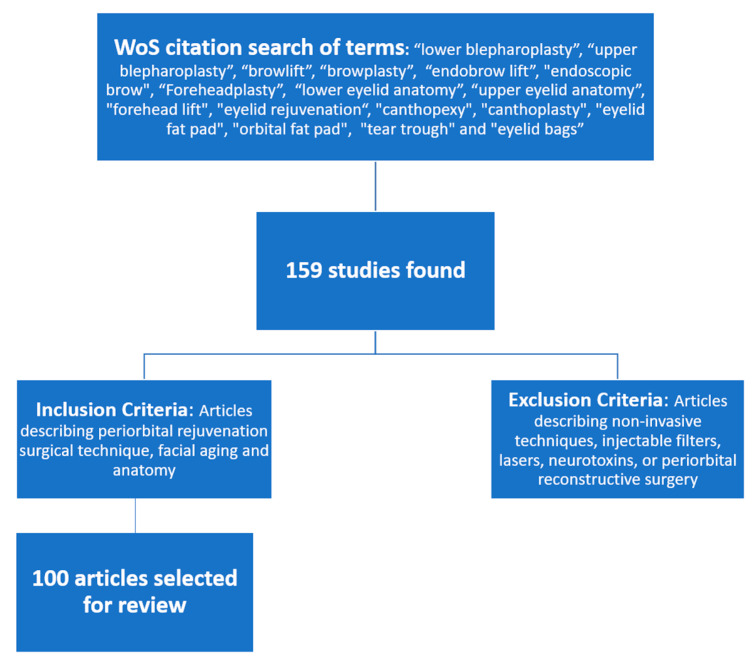
Methods flow chart. Abbreviations: WoS = Web of Science.

The Web of Science ^TM^ citation index was used to identify the 100 most-cited articles related to the topic of periorbital rejuvenation. Articles that were published in the English language from January 1989 to April 2020 were included in the study. The last 3 decades were selected as the timeframe of our study to ensure that the articles included were fundamental yet relevant to current periorbital surgical techniques. The terms “lower blepharoplasty”, “upper blepharoplasty”, “browlift”, “browplasty”, “endobrow lift”, “endoscopic brow”, “Foreheadplasty”, “lower eyelid anatomy”, “upper eyelid anatomy”, “forehead lift”, “eyelid rejuvenation”, “canthopexy”, “canthoplasty”, “eyelid fat pad”, “orbital fat pad”, “tear trough”, and “eyelid bags” were used in the citation search. The Web of Science Core Collection database was used in the search. A manual review of the initial 159 studies identified was performed. Articles that described non-invasive techniques, injectable fillers, lasers, neurotoxins, or periorbital reconstructive surgery were excluded. Articles describing periorbital rejuvenation-related surgical techniques, facial aging, and anatomy were included. After identifying the articles meeting the inclusion criteria, the top 100 were chosen to be highlighted in this article (Figure 1). In addition to citation count, the articles were further categorized by publication year, publishing journal, corresponding author’s primary specialty, focus of article, corresponding author’s country of residence, type of study, and level of evidence. The level of evidence was determined based on the methods described by Sullivan et al. [17]. 

## 3. Results

**Figure 2:**
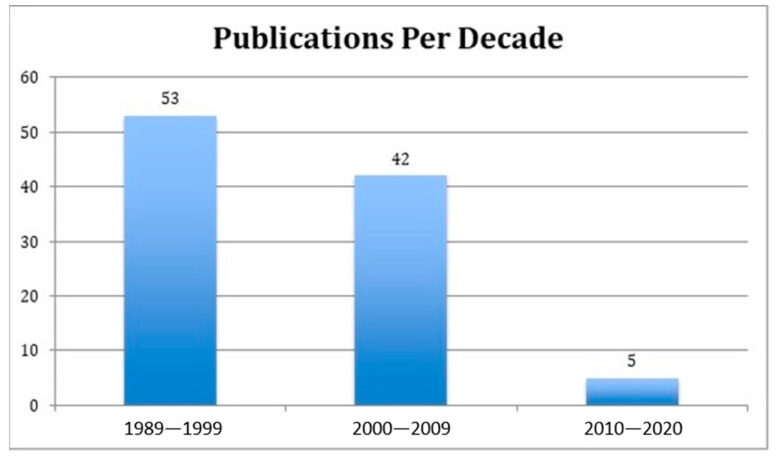
Categorization of the 100 most-cited articles by their publication decade.

**Figure 3:**
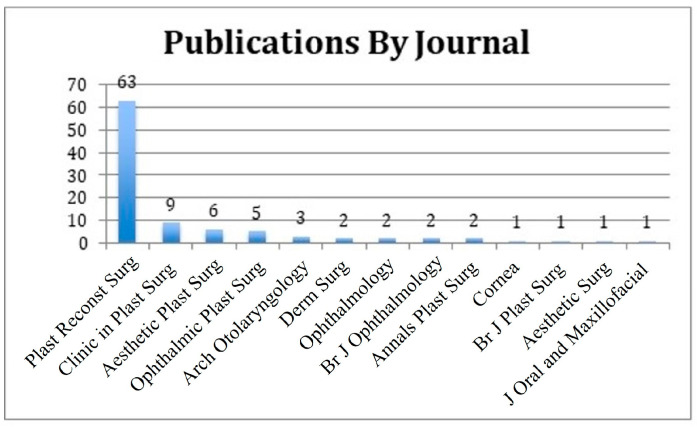
Categorization of the 100 most-cited articles by journal of publication. Abbreviations: Plast = plastic; Reconst = reconstructive; Surg = surgery, Derm = dermatologic; Br = British; J = journal.

**Figure 4:**
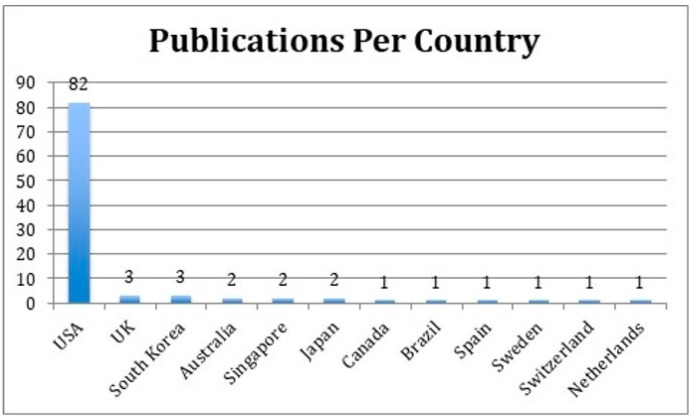
Categorization of the 100 most-cited articles by country of residence of the corresponding author.

**Figure 5:**
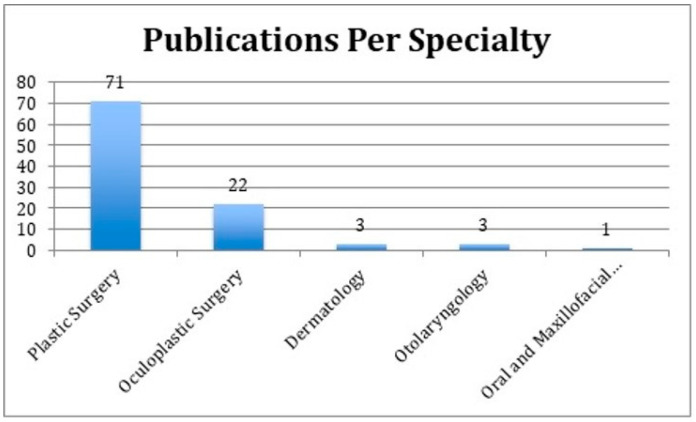
Categorization of the 100 most-cited articles by surgical specialty of the corresponding author.

The initial search was limited to 159 articles. Then, the abstracts of the most-cited 159 articles were individually reviewed. The 100 most-cited articles on periorbital rejuvenation are listed in this study (Table 1). The 100 most-cited articles were published between 1989 and 2020, spanning a 30-year period. The mean number of citations per article was 75 (standard deviation—SD: 42). There was a higher prevalence of articles published between 1989 and 1999 (*n* = 53) than later decades (Figure 2). Most articles (81%) were published in plastic surgery journals, of which 78% were published in the *Plastic and Reconstructive Surgery Journal* (*n* = 63) and originated from the United States (*n* = 82, 82%) (Figure 3 and Figure 4). Plastic and reconstructive surgery was the primary specialty of the corresponding authors in 71% of articles (*n* = 71), followed by oculoplastic surgery (*n* = 22) (Figure 5).

**Figure 6:**
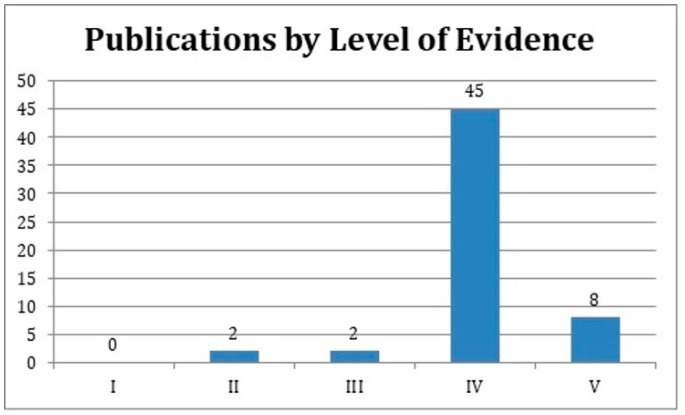
The levels of evidence of publications classified as clinical studies.

Of the 100 articles, 69 described surgical techniques, followed by anatomy (*n* = 22) and aging (*n* = 9). Clinical studies encompassed 57% of the top 100 most-cited articles, followed by review articles (*n* = 29) and basic science studies (*n* = 14). Clinical articles were classified according to their Level of Evidence. None of the studies provided level I evidence. Two studies [18] qualified as providing level II evidence, and two articles [19,20] provided level III evidence. Of the remaining 57 most-cited clinical studies, 45 studies (79%) provided level IV and 8 provided level V evidence (14%) (Figure 6).

**Table 1:** Details of the 100 Most-Cited Articles.

Rank	Corresponding Author	Title	Year	Journal	Citations	Author’s Specialty	Country	Focus of Article	Study Type	Level of Evidence
1	Pessa, J.E. [21]	The fat compartments of the face: anatomy and clinical implications for cosmetic surgery	2007	*Plast. Reconst. Surg*.	333	PRS	USA	Anatomy	Basic science	
2	Hamra, S.T. [22]	Arcus marginalis release and orbital fat preservation in midface rejuvenation	1995	*Plast. Reconst. Surg*.	224	PRS	USA	Technique	Clinical	IV
3	Lambros, V. [23]	Observations on periorbital and midface aging	2007	*Plast. Reconst. Surg*.	204	PRS	USA	Aging	Clinical	IV
4	Knize, D.M. [24]	An anatomically based study of the mechanism of eyebrow ptosis	1996	*Plast. Reconst. Surg*.	175	PRS	USA	Anatomy	Basic science	
5	Isse, N.G. [25]	Endoscopic facial rejuvenation—endoforehead, the functional lift—case-reports	1994	*Aesthetic Plast. Surg*.	166	PRS	USA	Technique	Clinical	V
6	Hester, T.R. [26]	Evolution of technique of the direct transblepharoplasty approach for the correction of lower lid and midfacial aging: maximizing results and minimizing complications in a 5-year experience	2000	*Plast. Reconst. Surg*.	128	PRS	USA	Technique	Clinical	IV
7	Hamra, S.T. [27]	The zygorbicular dissection in composite rhytidectomy: an ideal midface plane	1998	*Plast. Reconst. Surg*.	128	PRS	USA	Technique	Clinical	V
8	Mendelson, B.C. [16]	Surgical anatomy of the ligamentous attachments of the lower lid and lateral canthus	2002	*Plast. Reconst. Surg*.	127	PRS	Australia	Anatomy	Basic science	
9	Hykin, P.G. [18]	Age-related morphological changes in lid margin and meibomian gland anatomy	1992	*Cornea*	121	OPHT	UK	Aging	Clinical	II
10	Ramirez, O.M. [28]	Endoscopic techniques in facial rejuvenation—an overview .1.	1994	*Aesthetic Plast. Surg*.	120	PRS	USA	Technique	Review	
11	Baylis, H.I. [29]	Transconjunctival lower eyelid blepharoplasty—technique and complications	1989	*Ophthalmology*	114	OPHT	USA	Technique	Clinical	IV
12	Hamra, S.T. [30]	The role of orbital fat preservation in facial aesthetic surgery—a new concept	1996	*Clinic in Plast. Surg*.	113	PRS	USA	Technique	Clinical	V
13	Goldberg, R.A. [31]	Transconjunctival orbital fat repositioning: transposition of orbital fat pedicles into a subperiosteal pocket	2000	*Plast. Reconst. Surg*.	111	OPHT	USA	Technique	Clinical	IV
14	Jeong, S. [32]	The Asian upper eyelid—an anatomical study with comparison to the Caucasian eyelid	1999	*Arch. Opthalmol*.	102	OPHT	South Korea	Anatomy	Basic science	
15	Codner, M.A. [33]	Primary transcutaneous lower blepharoplasty with routine lateral canthal support: a comprehensive 10-year review	2008	*Plast. Reconst. Surg*.	99	PRS	USA	Technique	Clinical	IV
16	Pessa, J.E. [34]	Changes in ocular globe-to-orbital rim position with age: implications for aesthetic blepharoplasty of the lower eyelids	1999	*Aesthetic Plast. Surg*.	99	PRS	USA	Aging	Clinical	IV
17	Vasconez, L.O. [35]	Endoscopic techniques in coronal brow lifting	1994	*Plast. Reconst. Surg*.	97	PRS	USA	Technique	Clinical	IV
18	Fagien, S. [36]	Algorithm for canthoplasty: the lateral retinacular suspension: a simplified suture canthopexy	1999	*Plast. Reconst. Surg*.	95	OPHT	USA	Technique	Review	
19	Van Den Bosch, W.A. [37]	Topographic anatomy of the eyelids, and the effects of sex and age	1999	*Br* *. J* *. Ophthalmol.*	94	OPHT	Netherlands	Anatomy	Clinical	IV
20	Patipa, M. [38]	The evaluation and management of lower eyelid retraction following cosmetic surgery	2000	*Plast. Reconst. Surg*	89	OPHT	USA	Technique	Review	
21	Kikkawa, D.O. [39]	Relations of the superficial musculoaponeurotic system to the orbit and characterization of the orbitomalar ligament	1996	*Ophthalmic Plast. Reconst. Surg*.	89	OPHT	USA	Aging	Basic Science	
22	Jelks, G.W. [40]	The inferior retinacular lateral canthoplasty: a new technique	1997	*Plast. Reconst. Surg*.	88	PRS	USA	Technique	Clinical	V
23	Rohrich, R.J. [41]	The anatomy of suborbicularis fat: implications for periorbital rejuvenation	2009	*Plast. Reconst. Surg*.	86	PRS	USA	Anatomy	Basic Science	
24	Little, J.W. [42]	Three-dimensional rejuvenation of the midface: volumetric resculpture by malar imbrication	2000	*Plast. Reconst. Surg*.	86	PRS	USA	Technique	Clinical	IV
25	Hamra, S.T. [43]	Repositioning the orbicularis oculi muscle in the composite rhytidectomy	1992	*Plast Reconst Surg*	86	PRS	USA	Technique	Review	
26	Gunter, J.P. [44]	Aesthetic analysis of the eyebrows	1997	*Plast Reconst Surg*	85	PRS	USA	Anatomy	Clinical	IV
27	Flowers, R.S. [45]	Canthopexy as a routine blepharoplasty component	1993	*Clin. Plast. Surg*.	84	PRS	USA	Technique	Review	
28	Mullins, J.B. [46]	Complications of the transconjunctival approach—a review of 400 cases	1997	*Arch. Otoloaryngol.-H N Surg*.	82	ENT	USA	Technique	Clinical	IV
29	Aiache, A.E. [47]	The suborbicularis oculi fat pads—an anatomic and clinical-study	1995	*Plast. Reconst. Surg*.	82	PRS	USA	Technique	Clinical	V
30	McKinney, P. [48]	Criteria for the forehead lift	1991	*Aesthetic Plast. Surg*.	82	PRS	USA	Technique	Clinical	IV
31	Rohrich, R.J. [49]	Current concepts in aesthetic upper blepharoplasty	2004	*Plast. Reconst. Surg*.	78	PRS	USA	Technique	Review	
32	Thorne, C.H. [50]	The tear trough and lid/cheek junction: anatomy and implications for surgical correction	2009	*Plast. Reconst. Surg*.	77	PRS	USA	Anatomy	Basic Science	
33	Richard, M.J. [51]	Analysis of the anatomic changes of the aging facial skeleton using computer-assisted tomography	2009	*Ophthalmic Plast. Reconst. Surg*.	75	OPHT	USA	Aging	Clinical	IV
34	Knize, D.M. [52]	Transpalpebral approach to the corrugator supercilii and procerus muscles	1995	*Plast. Reconst. Surg*.	74	PRS	USA	Technique	Clinical	IV
35	Barton, F.E. [53]	Fat extrusion and septal reset in patients with the tear trough triad: a critical appraisal	2004	*Plast. Reconst. Surg*.	73	PRS	USA	Technique	Clinical	IV
36	McCord, C.D. [54]	Redraping the inferior orbicularis arc	1998	*Plast. Reconst. Surg*.	73	OPHT	USA	Technique	Review	
37	Wong, C.-H. [55]	The tear trough ligament: anatomical basis for the tear trough deformity	2012	*Plast. Reconst. Surg*.	71	PRS	Singapore	Anatomy	Basic Science	
38	Zarem, H.A. [56]	Expanded applications for transconjunctival lower lid blepharoplasty	1991	*Plast. Reconst. Surg*.	70	PRS	USA	Technique	Review	
39	Meyer, D.R. [57]	Anatomy of the orbital septum and associated eyelid connective tissues—implications for ptosis surgery	1991	*Ophthalmic Plast. Reconst. Surg*.	70	OPHT	USA	Anatomy	Basic Science	
40	McCord, C.D. [58]	Browplasty and browpexy—an adjunct to blepharoplasty	1990	*Plast. Reconst. Surg*.	70	OPHT	USA	Technique	Clinical	V
41	Goldberg, R.A. [59]	What causes eyelid bags? analysis of 114 consecutive patients	2005	*Plast Reconst Surg*.	69	OPHT	USA	Anatomy	Clinical	IV
42	Hass, A.N. [60]	Incidence of post-blepharoplasty orbital hemorrhage and associated visual loss	2004	*Ophthalmic Plast. Reconst. Surg*.	68	OPHT	USA	Technique	Clinical	IV
43	Hamra, S.T. [61]	The role of the septal reset in creating a youthful eyelid-cheek complex in facial rejuvenation	2004	*Plast* *. Reconst* *. Surg* *..*	68	PRS	USA	Technique	Review	
44	Fagien, S. [62]	Advanced rejuvenative upper blepharoplasty: enhancing aesthetics of the upper periorbita	2002	*Plast. Reconst. Surg*.	67	OPHT	USA	Technique	Review	
45	Knize, D.M. [63]	Limited-incision forehead lift for eyebrow elevation to enhance upper blepharoplasty	1996	*Plast. Reconst. Surg*.	67	PRS	USA	Technique	Clinical	IV
46	Ramirez, O.M. [64]	Three-dimensional endoscopic midface enhancement: A personal quest for the ideal cheek rejuvenation	2002	*Plast. Reconst. Surg*.	66	PRS	USA	Technique	Clinical	IV
47	Mendelson, B.C. [65]	Surgical anatomy of the midcheek: Facial layers, spaces, and the midcheek segments	2008	*Clin. in Plast. Surg*.	64	PRS	Australia	Anatomy	Review	
48	Park, D.H. [66]	Anthropometry of Asian eyelids by age	2008	*Plast. Reconst. Surg*.	63	PRS	South Korea	Aging	Clinical	IV
49	Freund, R.M. [67]	Correlation between brow lift outcomes and aesthetic ideals for eyebrow height and shape in females	1996	*Plast. Reconst. Surg*.	63	PRS	USA	Technique	Clinical	IV
50	Baker, D.C. [19]	Endoscopic brow lift: a retrospective review of 628 consecutive cases over 5 years	2003	*Plast. Reconst. Surg*.	62	PRS	USA	Technique	Clinical	III
51	Byrd, H.S. [68]	The deep temporal lift: a multiplanar, lateral brow, temporal, and upper face lift	1996	*Plast. Reconst. Surg*.	61	PRS	USA	Technique	Basic science	
52	McGraw, B.L. [69]	Post-blepharoplasty ectropion—prevention and management	1991	*Arch Otoloaryngol. H N Surg*.	61	ENT	Canada	Technique	Clinical	IV
53	Janis, J.E. [70]	Anatomy of the corrugator supercilii muscle: part i. corrugator topography	2007	*Plast. Reconst. Surg*.	60	PRS	USA	Anatomy	Basic Science	
54	Trepsat, F. [71]	Periorbital rejuvenation combining fat grafting and blepharoplasties	2003	*Aesthetic Plast. Surg*.	60	PRS	Switzerland	Technique	Review	
55	Daniel, R.K. [72]	Endoscopic forehead lift: an operative technique	1996	*Plast. Reconst. Surg*.	60	PRS	USA	Technique	Clinical	IV
56	Carraway, J.H. [73]	The prevention and treatment of lower lid ectropion following blepharoplasty	1990	*Plast. Reconst. Surg*.	60	PRS	USA	Technique	Review	
57	McCord, C.D. [74]	Lateral canthal anchoring	2003	*Plast. Reconst. Surg*.	59	OPHT	USA	Technique	Review	
58	Rohrich, R.J. [75]	Evolving fixation methods in endoscopically assisted forehead rejuvenation: controversies and rationale	1997	*Plast. Reconst. Surg*.	59	PRS	USA	Technique	Review	
59	Jones, B.M. [76]	Endoscopic brow lift: a personal review of 538 patients and comparison of fixation techniques	2004	*Plast. Reconst. Surg*.	58	PRS	UK	Technique	Clinical	IV
60	May, J.W. [77]	Retro-orbicularis oculus fat (roof) resection in aesthetic blepharoplasty—a 6-year study in 63 patients	1990	*Plast. Reconst. Surg*.	58	PRS	USA	Technique	Clinical	IV
61	Lisman, R.D. [78]	Blepharoplasty complications	2010	*Plast. Reconst. Surg*.	57	OPHT	USA	Technique	Review	
62	Elkwood, A. [79]	National plastic surgery survey: brow lifting techniques and complications	2001	*Plast. Reconst. Surg*.	57	PRS	USA	Technique	Review	
63	Paul, M.D. [80]	The evolution of the brow lift in aesthetic plastic surgery	2001	*Plast. Reconst. Surg*.	57	PRS	USA	Technique	Review	
64	Hamra, S.T. [81]	Frequent face lift sequelae: hollow eyes and the lateral sweep: cause and repair	1998	*Plast. Reconst. Surg*.	56	PRS	USA	Technique	Review	
65	Loeb, R. [82]	Naso-jugal groove leveling with fat tissue	1993	*Clin. Plast. Surg*.	56	PRS	Brazil	Technique	Review	
66	Connell, B.F. [83]	The forehead lift—techniques to avoid complications and produce optimal results	1989	*Aesthetic Plast. Surg*.	56	PRS	USA	Technique	Review	
67	Rohrich, R.J. [84]	The five-step lower blepharoplasty: blending the eyelid-cheek junction	2011	*Plast. Reconst. Surg*.	55	PRS	USA	Technique	Clinical	IV
68	Flowers, R.S. [85]	Upper blepharoplasty by eyelid invagination—anchor blepharoplasty	1993	*Clin. Plast. Surg.*	55	PRS	USA	Technique	Clinical	V
69	Wong, C.H. [86]	Facial soft-tissue spaces and retaining ligaments of the midcheek: defining the premaxillary space	2013	*Plast. Reconst. Surg*.	53	PRS	Singapore	Anatomy	Basic Science	
70	Jelks, G.W. [87]	Evolution of the lateral canthoplasty: techniques and indications	1997	*Plast. Reconst. Surg*.	53	PRS	USA	Technique	Clinical	IV
71	Goldberg, R.A. [88]	Eyelid anatomy revisited—dynamic high-resolution magnetic-resonance images of whitnall ligament and upper eyelid structures with the use of a surface coil	1992	*Arch* *. Opthalmo* *l.*	53	PRS	USA	Anatomy	Clinical	IV
72	Yaremchuk, M.J. [89]	Changes in eyebrow position and shape with aging	2009	*Plast. Reconst. Surg*.	52	PRS	USA	Aging	Clinical	IV
73	Lambros, V. [90]	Models of facial aging and implications for treatment	2008	*Clin. Plast. Surg*.	52	PRS	USA	Aging	Review	
74	Nahai, F. [91]	Transconjunctival blepharoplasty for upper and lower eyelids	2010	*Plast. Reconst. Surg*.	51	PRS	USA	Technique	Clinical	IV
75	Tyers, A.G. [92]	The direct brow lift: efficacy, complications, and patient satisfaction	2004	*Br. J. Ophthalmol*.	51	OPHT	UK	Technique	Clinical	IV
76	Patel, B.C.K. [93]	Management of post-blepharoplasty lower eyelid retraction with hard palate grafts and lateral tarsal strip	1997	*Plast. Reconst. Surg*.	51	OPHT	USA	Technique	Clinical	IV
77	Pessa, J.E. [94]	The orbicularis retaining ligament of the medial orbit: closing the circle	2008	*Plast. Reconst. Surg*.	50	PRS	USA	Anatomy	Basic Science	
78	Kawamoto, H.K. [95]	The tear “trouf” procedure: transconjunctival repositioning of orbital unipedicled fat	2003	*Plast. Reconst. Surg*.	50	PRS	USA	Technique	Clinical	IV
79	Ramirez, O.M. [96]	Endoscopically assisted biplanar forehead lift	1995	*Plast. Reconst. Surg*.	50	PRS	USA	Technique	Clinical	IV
80	Troilius, C. [97]	Subperiosteal brow lifts without fixation	2004	*Plast. Reconst. Surg*.	49	PRS	Sweden	Technique	Clinical	II
81	Furnas, D.W. [98]	Festoons, mounds, and bags of the eyelids and cheek	1993	*Clin. Plast. Surg*.	49	PRS	USA	Anatomy	Review	
82	Shore, J.W. [99]	Operative complications of the transconjunctival inferior fornix approach	1991	*Ophthalmology*	49	OPHT	USA	Technique	Clinical	V
83	De la Plaza, R. [100]	Supraperiosteal lifting of the upper 2/3 of the face	1991	*Br. J. Plast. Surg*.	49	PRS	Spain	Technique	Clinical	IV
84	De la Torre, J. [101]	Endoscopic forehead lift: review of technique, cases, and complications	2002	*Plast. Reconst. Surg*.	48	PRS	USA	Technique	Clinical	IV
85	Chung, K.Y. [102]	Infraorbital dark circles: definition, causes, and treatment options	2009	*Derm. Surg*.	47	Dermatology	South Korea	Technique	Review	
86	Moy, R.L. [103]	Objective changes in brow position, superior palpebral crease, peak angle of the eyebrow, and jowl surface area after volumetric radiofrequency treatments to half of the face	2004	*Derm. Surg*.	47	Dermatology	USA	Technique	Clinical	IV
87	Isse, N.G. [104]	Endoscopic forehead lift—evolution and update	1995	*Clin. Plast. Surg*.	47	PRS	USA	Technique	Clinical	IV
88	Hamra, S.T. [105]	A study of the long-term effect of malar fat repositioning in face lift surgery: short-term success but long-term failure	2002	*Plast. Reconst. Surg*.	45	PRS	USA	Technique	Clinical	IV
89	Starck, W.J. [106]	Objective evaluation of the eyelids and eyebrows after blepharoplasty	1996	*J. Oral Maxillofac. Surg*.	45	OMS	USA	Technique	Clinical	IV
90	Cook, T.A. [107]	The versatile midforehead lift	1989	*Arch. Otoloaryngol. H N Surg*.	45	ENT	USA	Technique	Clinical	IV
91	Knize, D.M. [108]	Anatomic concepts for brow lift procedures	2009	*Plast. Reconst. Surg*.	44	PRS	USA	Anatomy	Review	
92	Core, G.B. [109]	Endoscopic browlift	1995	*Clin. Plast. Surg*.	44	PRS	USA	Technique	Review	
93	Rudkin, G.H. [110]	Magnetic resonance imaging characterization of orbital changes with age and associated contributions to lower eyelid prominence	2008	*Plast. Reconst. Surg*.	43	PRS	USA	Aging	Clinical	IV
94	Carter, S.R. [20]	The Asian lower eyelid: A comparative anatomic study using high-resolution magnetic resonance imaging	1998	*Ophthalmic Plast. Reconst. Surg*.	43	OPHT	USA	Anatomy	Clinical	III
95	Kakizaki, H. [111]	Lower eyelid anatomy An update	2009	*Ann. Plast. Surg*.	42	OPHT	Japan	Anatomy	Review	
96	Paul, M.D. [112]	The evolution of the midface lift in aesthetic plastic surgery	2006	*Plast. Reconst. Surg*.	42	PRS	USA	Technique	Review	
97	Pessa, J.E. [113]	The malar septum: The anatomic basis of malar mounds and malar edema.	1997	*Aesthetic Surg. J.*	42	PRS	USA	Anatomy	Basic Science	
98	Ramirez, O.M. [114]	The anchor subperiosteal forehead lift	1995	*Plast. Reconst. Surg*.	42	PRS	USA	Technique	Clinical	IV
99	Guyuron, B. [115]	Corrugator supercilii muscle resection through blepharolplasty Incision	1995	*Plast. Reconst. Surg*.	42	PRS	USA	Technique	Clinical	IV
100	Kakizaki, H. [116]	Upper eyelid anatomy An update	2009	*Ann. Plast. Surg*.	41	OPHT	Japan	Anatomy	Review	

Abbreviations: PRS = plastic and reconstructive surgery; OPHT = ophthalmology; OMS = oral and maxillofacial surgery; ENT = ear, nose, and throat.

**Figure 7:**
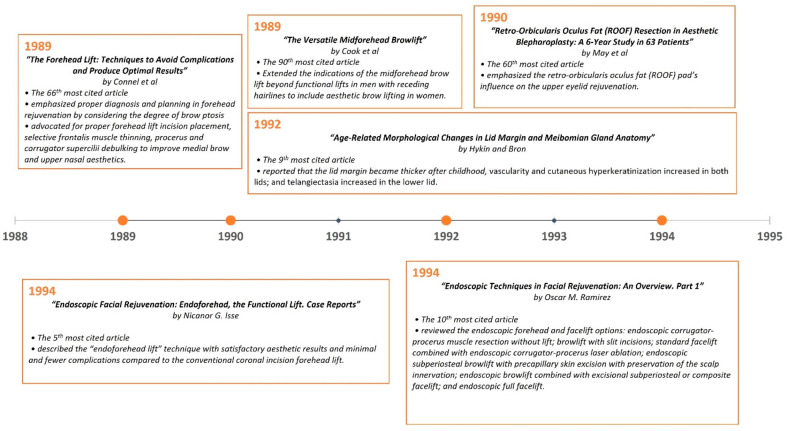
Most impactful articles from 1989 to 1994.

**Figure 8:**
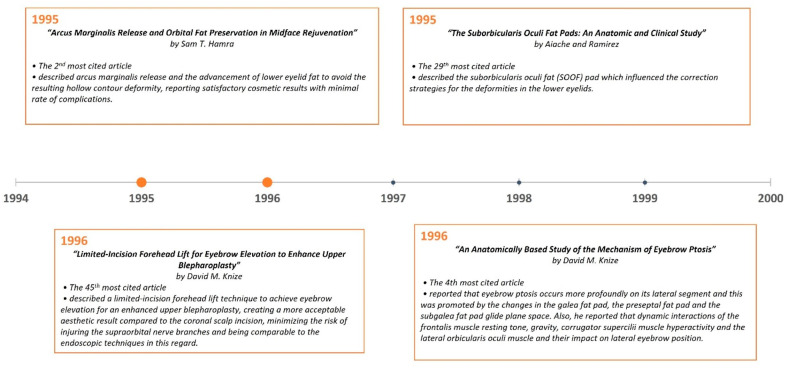
Most impactful articles from 1995 to 1999.

**Figure 9:**
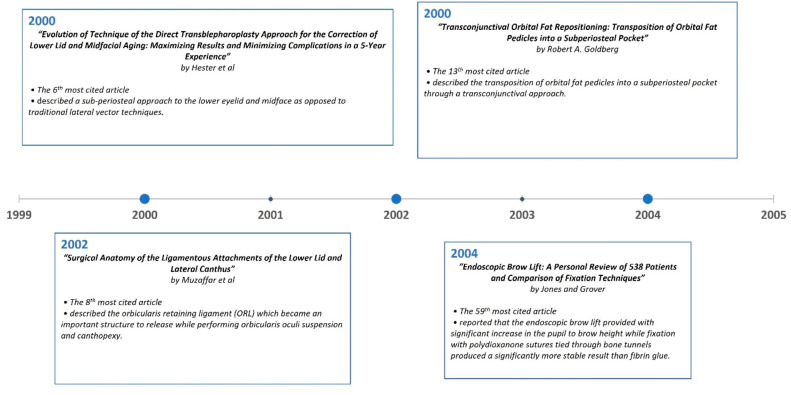
Most impactful articles from 2000 to 2004.

**Figure 10:**
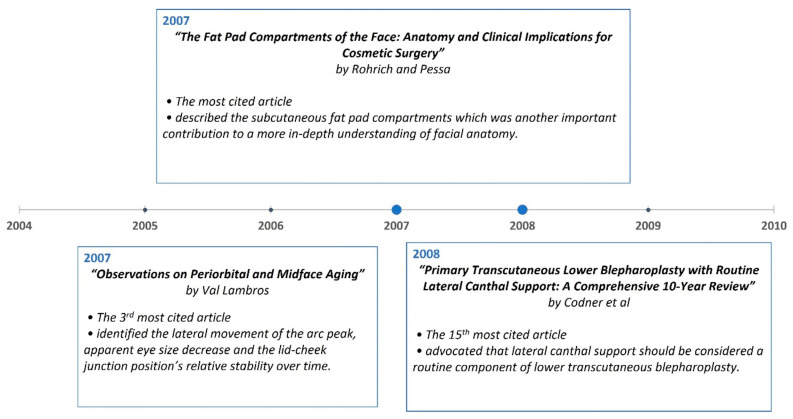
Most impactful articles from 2005 to 2009.

**Figure 11:**
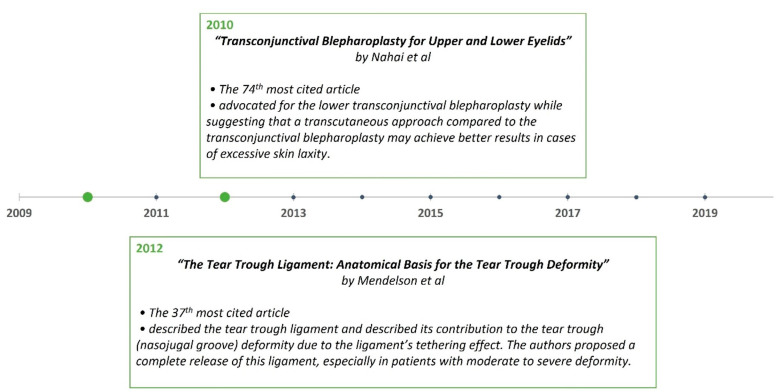
Most impactful articles from 2010 to 2020.

**Figure 12:**
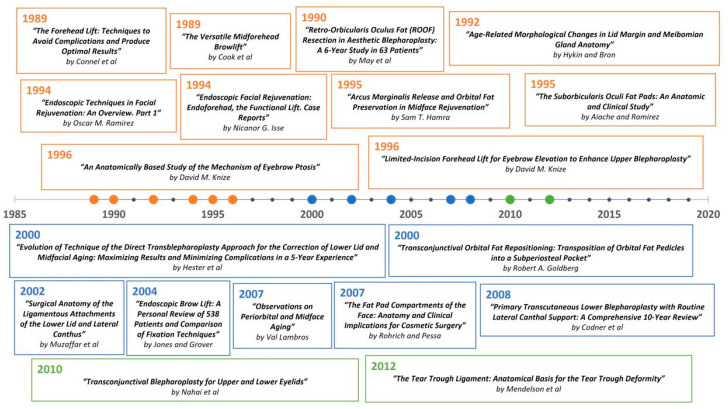
Summative timeline of the most impactful articles on the topic of periorbital rejuvenation from 1989 through 2020.

A timeline of the articles was created to better visualize the evolution of periorbital rejuvenation surgery techniques (Figure 7, Figure 8, Figure 9, Figure 10, Figure 11 and Figure 12). The most clinically relevant articles within each 5-year period are featured in the timeline.

## 4. Discussion

Facial aging is a result of changes in the five lamellar structures of the face and the underlying facial bony skeleton. Throughout life, there is apparent descent, atrophy, or hypertrophy of certain compartments within the face that make it appear more aged [23,34,117]. Aging is manifested in the lateral translation of the orbits, glabellar protrusion, the expansion of the supraorbital ridges, the deepening and lateral expansion of the cheeks, the three-dimensional enlargement of the nose, and an increase in chin prominence, as described by Enlow [118] and Mendelson and Wong [119]. The interplay between the bony skeleton, supporting ligaments, fat compartments, and facial mimetic muscles is influenced by physiological, genetic, and environmental factors [120]. These factors involve bone remodeling and functional effects of the surrounding muscles’ action (i.e., the effects of chronic orbicularis oculi contraction on lateral brow position). The youthful eye is characterized by an almond-shaped palpebral fissure with a slight upward slope from the medial to lateral canthus. Concerning periorbital aging, Lambros [23,121] noted “the eyes seem to get smaller as one ages, the entire lid aperture gets smaller because the lower lid rises, the upper lid falls, the lid gets shorter from the side, and the fat pads enlarge” [26,122]. Therefore, surgeons treating periorbital aging should consider all of these structures and the accompanying changes to achieve optimal rejuvenation. The improvement in the anatomical understanding of periorbital structures over the last 3 decades has contributed to a better understanding of the aging process and has enhanced the surgical strategies used to achieve more youthful and attractive eyes [10,21,47,55,117,123,124,125,126].

### 4.1. Evolution of Periorbital Rejuvenation over the Last 3 Decades

Periorbital rejuvenation has evolved to include a multitude of approaches to addressing aging-related changes of the orbit such as skin excision, SMAS re-draping, orbicularis oculi muscle repositioning, fat pad reduction or transposition, the release of the orbicularis-retaining ligament (ORL), and micro/nano fat grafting [26,31,43,47,127]. A comprehensive approach to upper eyelid rejuvenation includes the brow and forehead. Similarly, for lower lid rejuvenation, the midface is involved [128]. 

In 1989, Cook et al. [107] reported their experience in extending the indications of the midforehead brow lift beyond functional lifts in men with receding hairlines to include aesthetic brow lifting in women. The authors described their surgical technique, which included staggering midforehead elliptical excisions, the undermining of the inferior forehead skin, and the placement of suspension sutures in the mid and lateral brow. Good results among 52 female patients were reported. This study was ranked 90th among the top 100 most-cited articles. In the same year, Connell et al. [83] reported their approach to forehead lifting and emphasized proper diagnosis and planning with respect to forehead rejuvenation by considering the degree of brow ptosis associated with the upper eyelid skin’s laxity. They advocated for the proper placement of forehead lift incisions, selective frontalis muscle thinning, and procerus and corrugator supercilii debulking to improve medial brow and upper nasal aesthetics. In 1994, Isse [25] described his endoscopic “endoforehead lift” technique in a total of 61 cases, and his article became the 5th most-cited article. The author reported that he was able to achieve satisfactory cosmetic results with minimal and fewer complications when compared to the conventional coronal incision forehead lift. That same year, Ramirez [28] reviewed different endoscopic forehead and facelift options (the 10th most-cited article). These included endoscopic corrugator-procerus muscle resection without a lift, a browlift with slit incisions, a standard facelift combined with endoscopic corrugator-procerus laser ablation, an endoscopic subperiosteal browlift with precapillary skin excision and the preservation of the scalp’s innervation, an endoscopic browlift combined with an excisional subperiosteal or composite facelift, and an endoscopic full facelift. In 1996, Knize [63] described his limited-incision forehead lift technique to achieve eyebrow elevation for an enhanced upper blepharoplasty. In this technique, the author used temporal scalp incisions of only 4.5 to 5 cm in length while also performing a transpalpebral resection of the corrugator supercilii muscles and a transection of the procerus muscle. This created a more acceptable aesthetic result compared to the coronal scalp incision, thus minimizing the risk of injuring the supraorbital nerve branches and being comparable to the endoscopic techniques in this regard. This article has become the 45th most-cited article related to periorbital rejuvenation. By the early 2000s, the endoscopic brow/forehead lift had gained significant popularity [19,35,75,76]. In 2004, Jones and Grover [76] reported their experience with 538 endoscopic brow lift cases and compared the outcomes of two different fixation techniques: fibrin glue versus polydioxanone sutures tied through bone tunnels. The authors found that the endoscopic brow lift provided a significant increase in the pupil to brow height while fixation with polydioxanone sutures tied through bone tunnels produced a significantly more stable result than fibrin glue.

In 1995, Hamra [22] described arcus marginalis release and the advancement of lower eyelid fat as an alternative to its excision to avoid the resulting hollow contour deformity, which is now synonymous with “the operated appearance”. In his study of 152 cases, which is the second most-cited article, Hamra reported satisfactory results with a minimal rate of complication. In 2000, Goldberg [31] described the transposition of orbital fat pedicles into a subperiosteal pocket through a transconjunctival approach. That same year, Hester et al. [26] published a retrospective review of 757 patients who underwent direct trans-lower eyelid blepharoplasty to correct midfacial aging. In this sixth most-cited paper (*n* = 128), the authors described a sub-periosteal approach to the lower eyelid and midface as opposed to traditional lateral vector techniques. Two years later, Muzaffar et al. [16] described the ORL, which became an important structure to release while performing orbicularis oculi suspension (re-draping) and canthopexy. In 2012, Mendelson et al. [55] described the tear trough ligament, a true osteocutaneous ligament, and its contribution to tear trough (nasojugal groove) deformity due to the ligament’s tethering effect. The authors proposed a complete release of this ligament, especially in patients with moderate to severe deformity. 

As a topic of continued debate, it is also worth discussing the popularity and preference for the transcutaneous and transconjunctival approaches to lower blepharoplasty. Both techniques have been represented in the top 100 cited article list but there was a greater number of articles advocating the transconjunctival approach (*n* = 5 versus *n* = 1). In their study in 2008, Codner et al. [33] reviewed their experience with primary lower transcutaneous blepharoplasty via a subciliary skin incision in 264 patients over 10 years, which was the 15th most-cited article included in our study. The authors reported that nine (3.5%) patients had eyelid malposition that required operative correction while one (0.4%) patient had an orbital hematoma. They concluded that lateral canthal support should be considered a routine component of lower transcutaneous blepharoplasty. In 2010, Pacella et al. [91] reviewed the anatomy, indications, and outcomes of lower transconjunctival blepharoplasty in their article (the 74th most-cited article). In their senior author’s personal experience (Dr. Foad Nahai) with 300 lower lid blepharoplasties between 1992 and 1995, the complication rate for the transconjunctival group was 5% (6 out of 120 patients) versus 13% (24 out of 180 patients) for the transcutaneous group. In the transconjunctival group, there was no lid retraction, which was a complication experienced by 3.3% (*n* = 6) in the transcutaneous group. The authors concluded that the transconjunctival lower blepharoplasty was a safe and effective procedure for periorbital rejuvenation. They also noted that in cases of excessive skin laxity, a transcutaneous approach compared to the transconjunctival blepharoplasty may achieve better results with the addition of lateral canthoplasty and/or lateral canthal anchoring procedure to minimize the risk of lower-lid malposition. The importance of the proper assessment and diagnosis of factors that may contribute to lower-lid malposition was highlighted by Jelks et al. [40,87,129]. As such, lateral canthoplasty for managing potential lower-lid malposition after blepharoplasty has become routine practice in contemporary lower blepharoplasty. 

As emphasized in this article, the studies on facial aging and anatomy have impacted periorbital rejuvenation strategies significantly. In 1992, Hykin and Bron [18] studied the age-related changes in the eyelid margin in 80 subjects, and this article has become the ninth most-cited article (*n* = 121). In the study, it was reported that with aging, the lid margin became thicker after childhood, lid margin vascularity and cutaneous hyperkeratinization increased in both lids, and telangiectasia increased in the lower lid. The description of suborbicularis orbital fat (SOOF) by Aiache and Ramirez [47] in 1995 also influenced the correction strategies for deformities in the lower eyelids, just as retroorbicularis oculus fat (ROOF) influenced the upper eyelid rejuvenation technique pioneered by Owsley [130] and May [77]. In the following year, Knize’s [24] anatomical study on 20 (40 half-head) fresh cadavers was published and has since become the 4th most-cited article (*n* = 175). The author reported that eyebrow ptosis occurs more profoundly on the brow’s lateral segment, and this was promoted by the changes in the galeal fat pad, the preseptal fat pad, and the subgaleal fat pad glide plane space. In addition, he described the impact of the dynamic interactions between the frontalis muscle’s resting tone and gravity, and the corrugator supercilii and the lateral orbicularis oculi muscles’ hyperactivity on lateral eyebrow position. In 2007, the cadaveric study by Rohrich and Pessa [21] described the subcutaneous fat pad compartments, constituting another important contribution to a more in-depth understanding of facial anatomy. This is the most-cited article of the articles related to periorbital rejuvenation within the last 3 decades. Another important publication that impacted rejuvenation strategies was Lambros’ [23] observational study published in the same year, which has become the third most-cited article (*n* = 204). He compared the 10- to 50-year-old photographs of 130 subjects with their recently taken follow-up photographs to assess the effects of aging on the face. The interesting findings from this study included the lateral movement of the arc peak, the apparent decrease in eye size, and the relative stability of the position of the lid–cheek junction over time. 

Although not the subject of this article, injectables play an important role in modern periorbital rejuvenation. In 1981, bovine collagen became the first agent that was approved for cosmetic injection by the FDA [131]. The introduction of hyaluronic acid in 2003 initiated a new era for non-surgical peri-orbital and facial rejuvenation. According to statistics released by the American Society of Plastic Surgeons (ASPS), 79.5% of 2,676,970 soft tissue filler procedures performed in 2018 used hyaluronic acid fillers [132]. These fillers are currently used widely by a multitude of practitioners across multiple specialties with satisfactory aesthetic results; however, their relatively short-lived effect is the main drawback compared to these surgical techniques. Another important factor, which is not accounted for in the cited articles but impacts eyelid and brow rejuvenation trends nonetheless, is society’s evolving perception of beauty in each era and the influence of pop culture and social media on such trends. What Westmore postulated as the ideal female brow position in the 1980s has now been replaced by the more lateral position of the brow’s peak closer to the lateral canthus [133].

As we have summarized, the current surgical techniques of periorbital rejuvenation have evolved as our understanding of facial has anatomy progressed. More conservative fat and muscle excisions with which to prevent the “operated” appearance and post-operative complications dominate our current approaches to peri-orbital rejuvenation [49]. Twenty-one percent of the one hundred most-cited articles within the last 31 years focused on periorbital anatomy and its clinical relevance to periorbital aesthetic surgery. This list of articles offers a comprehensive compilation of studies to readers interested in advancing their knowledge of periorbital rejuvenation. 

This study is not without limitations. There is potential for citation bias contributing to the citation rankings in this study. Authors may tend to select references that support their conclusions [134]. Self-citation can also play a role in citation bias [135]. Furthermore, the number of citations in the last decade remained the lowest among the 3 decades reported (Figure 2). This does not mean that these articles were of lesser importance or had a lower impact on practice changes; rather, it reflects the time factor required for these articles to be cited. Lastly, as more articles on the same topic are published, the authors have a larger pool of references from which to cite, thus generating a dilution effect concerning the articles published in later years compared to the older citations. 

### 4.2. Current Approaches to Brow and Eyelid Rejuvenation

There are three surgical approaches to browlift: trans-blepharoplasty brow lift, direct brow lift, and trans-forehead brow/forehead lift. The latter two have the longest-lasting effect [136]. Direct browlifts are most often used in patients with brow ptosis due to a nerve injury, such as those with Bell’s Palsy, but they are also good options for men with a receded hairline and women who desire only a lateral brow lift [137]. It is the most predictable method for browlift, as the incision is placed just above the superior end of the brow or along a rhytid near the brow. In addition, the degree of lift one can expect post-operatively is proportional to the amount of tissue removed [138]. However, scars are a major concern with respect to direct brow lift and there is currently a move towards more minimally invasive approaches [136,139,140]. 

**Figure 13:**
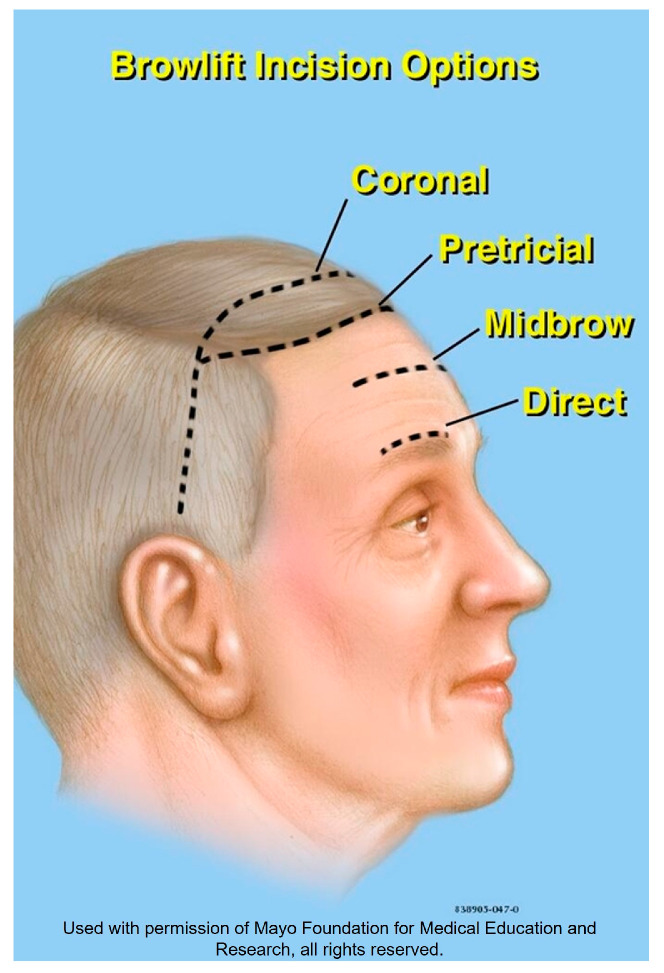
Illustration demonstrating the different types of incisions used for browlift surgery.

The endoscopic approach is another technique that was popular in the last 3 decades and may still be practiced by some surgeons. However, other techniques, such as the gliding brow lift, are gaining more popularity. The gliding brow lift can elevate the brow without raising the forehead’s height [141]. One advantage of this technique is that it requires only two 3 mm scalp incisions in the frontotemporal area. The surgeon undermines directly above the frontalis and galea down to 1 cm below the eyebrows. A plane of subcutaneous tissue is elevated and then sutured to the frontalis and galea via a hemostatic net to maintain brow elevation. As with any new technique, there are early, middle, and late adopters, resulting in a variable lag time between the introduction of a surgical technique and publications supporting its safety or efficacy. Figure 13 demonstrates a summary of the types of incisions used in the different types of browlift. 

Upper blepharoplasty is often combined with browlift surgery. Current methods of upper eyelid blepharoplasty are more conservative and refrain from the removal of the orbicularis oculi and excessive orbital fat resection to prevent a hollowed-out appearance. Modern lower blepharoplasty has shifted from fat resection to volume redistribution or augmentation through fat transposition or micro and nano fat grafting [142,143]. While these techniques may be more popular in current practice, citations supporting their use may underreport their popularity.

There are two main surgical approaches to lower blepharoplasty: a transcutaneous and transconjunctival approach. Based on questionnaires sent out to members of the American Society of Ophthalmic Plastic and Reconstructive Surgery who perform blepharoplasty, more surgeons use the transconjunctival approach [144]. Some advantages of the transconjunctival approach include the absence of scarring following surgery, decreased recurrence of lower-lid bulging, and the avoidance of complications such as vertical lid shortening [145]. Fat repositioning and canthal suspension were frequently performed along with lower blepharoplasty [144]. Regarding fat repositioning, the planes of dissection are subperiosteal, supraperiosteal, and intra-SOOF. The complication rates in these three planes are low when performed by experienced surgeons, but there is a high learning curve. Dissecting the supraperiosteal plane runs the risk of injuring the blood vessels, while using the subperiosteal plane does not allow for the release of the ORL, tear trough, and lid–cheek junction. To address this, a new approach using the midcheek spaces for orbital fat repositioning has been proposed, offering improvements in terms of lower-lid fat and herniated orbital fat and a decreased prominence of the lid–cheek junction [146]. Additionally, combining lower eyelid surgery with midface rejuvenation surgery can result in greater cosmetic outcomes, depending on the degree of lower eyelid skin laxity, midface descent, and midface volume [147].

While women accounted for 85% of blepharoplasty procedures in the United States, a transconjunctival approach is preferred in men undergoing lower blepharoplasty, as their primary concern was found to be the formation of the deep palpebromalar groove and tear-trough deformity [148,149]. A transconjunctival approach permits easy access to the periorbital fat compartments for fat excision.

## 5. Conclusions

Over the last 3 decades, periorbital rejuvenation techniques have evolved in tandem with our knowledge of periorbital anatomy and aging. Studies focusing on periorbital anatomy, aging, and surgical techniques were the most-cited publications. An anatomically based approach that is customized for each patient and accounts for age-related changes in the periorbital structures is paramount in contemporary periorbital rejuvenation.

## Data Availability

Not applicable.
